# Selectivity of Inhibition of N-Succinyl-**l**,**l**-Diaminopimelic Acid Desuccinylase *in Bacteria*: The product of dapE-gene Is Not the Target of **l**-Captopril Antimicrobial Activity

**DOI:** 10.1155/2011/306465

**Published:** 2011-03-31

**Authors:** Narasimha Rao Uda, Marc Creus

**Affiliations:** Department of Chemistry, University of Basel, Spitalstrasse 51, Basel 4056, Switzerland

## Abstract

The emergence of bacterial strains that are resistant to virtually all currently available antibiotics underscores the importance of developing new antimicrobial compounds. N-succinyl-l,l-diaminopimelic acid desuccinylase (DapE) is a metallohydrolase involved in the meso-diaminopimelate (mDAP)/lysine biosynthetic pathway necessary for lysine biosynthesis and for building the peptidoglycan cell wall. Because DapE is essential for Gram-negative and some Gram-positive bacteria, DapE has been proposed as a good target for antibiotic development. Recently, l-captopril has been suggested as a lead compound for inhibition of DapE, although its selectivity for this enzyme target in bacteria remains unclear (Gillner et al. (2009)). Here, we tested the selectivity of l-captopril against DapE *in bacteria*. Since DapE knockout strains of gram-negative bacteria are viable upon chemical supplementation with mDAP, we reasoned that the antimicrobial activity of compounds targeting DapE should be abolished in mDAP-containing media. Although l-captopril had modest antimicrobial activity in *Escherichia coli* and in *Salmonella enterica*, to our surprise, inhibition of bacterial growth was independent both of mDAP supplementation and DapE over-expression. We conclude that DapE is not the main target of l-captopril inhibition in these bacteria. The methods implemented here will be useful for screening DapE-selective antimicrobial compounds directly in bacterial cultures.

## 1. Introduction

**Scheme 1:**
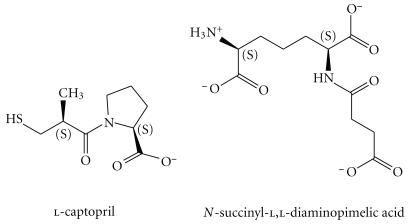
Structures of l-captopril and *N*-succinyl-l,l-diaminopimelic acid.

Most novel antibiotics that are developed are simply broad-spectrum, structural variants of a limited set of known bioactive compounds, many targeting the same enzymatic pathways. Consequently, the risk that pathogenic bacterial strains eventually evolve resistance against new antibiotics is very high [1, 2]. To help combat the serious problem of antibiotic resistance, it is imperative that new enzymatic targets are identified and that their specific inhibitors are developed. DapE, N-succinyl-l,l-diaminopimelic acid desuccinylase of the meso-diaminopimelate (mDAP)/lysine biosynthetic pathway of bacteria has been identified as an attractive potential antibiotic target [3]. DapE is a terminal enzyme for the hydrolysis of the N-succinyl-l,l-diaminopimelic acid (SDAP) (Scheme 1) into succinate and DAP [4]. Two of the products of this pathway (mDAP and lysine) are essential components of the cell: lysine is a protein-amino acid and DAP is a necessary component of the peptidoglycan cell wall of all Gram-negative and many Gram-positive bacteria [3, 5]. Since there is no enzyme in mammals similar to DapE, the inhibitors of DapE could potentially provide selective toxicity against bacteria and have little or no effect on humans. 

DapE is a homodimeric enzyme, with each monomer (41.6 KDa) containing two structural domains: a dimerization domain and a catalytic domain with a di-zinc active site [4, 6]. Sequence alignment of all known DapE enzymes, including of *E. coli* and *S. enterica,* with the structurally characterized DapE of *H. influenzae* and *Neisseria meningitides,* points toward the very strict conservation of all the amino acids that function as metal ligands and putative substrate binding sites [4, 7–9].

Strong inhibition of metalloenzymes has often been achieved by direct coordination of catalytic metals within the active site. Three examples of such inhibition employed successfully in clinical drugs are the sulphonamides (as carbonic anhydrase inhibitors) [10], suberoylanilide hydroxamic acid (SAHA) as histone deacetylase inhibitor [11], and l-captopril (Scheme 1), which was the first marketed antihypertension drug, targeting angiotensin I converting enzyme (ACE) [12]. Captopril binds to the catalytic zinc of ACE through coordination by a sulfhydryl group. Although captopril also shows some inhibitory activity towards other zinc metalloproteases, this is typically several orders of magnitude weaker than with ACE [12].

Recently, Gillner and colleagues [3] also identified captopril amongst the best inhibitors of *H. influenzae* DapE in a screen biased toward compounds containing zinc-binding groups (including thiols, carboxylic acids, boronic acids, phosphonates, and hydroxamates). Captopril was found to be a low-micromolar inhibitor of DapE (IC_50_ = 3.3 *μ*M, K_i_ = 1.8 *μ*M) and had antimicrobial activity *in bacteria* (against *E. coli*). However, here we present evidence that DapE is not the main target of l-captopril antimicrobial activity *in bacteria*.

## 2. Bacterial Strains, Plasmids, Media, and Other Materials

*Escherichia coli *XL1-Blue was purchased from Stratagene. *Salmonella enterica *serovar Typhimurium DapE knockout-strain (TN5911) and the plasmid, pCM655/DapE, were kindly provided by Prof. Miller [13]. E-medium [14] supplemented with 0.4% glucose and a 0.4 mM concentration of the appropriate amino acids (Bachem) was used as a minimal medium, and LB-medium was used as a rich medium. As a supplement, *meso*-diaminopimelate (mDAP) was added at 1 mM (Bachem). l-captopril was purchased from Sigma Aldrich. Sodium ampicillin, tetracycline and chloramphenicol were used at final concentrations of 60, 5, and 34 *μ*g/mL, respectively, when added to either liquid or solid medium. Liquid cultures were aerated by shaking on a rotary shaker (250 rpm), and all growth incubations were at 37°C for 19 hours. Electro competent cells of *S. enterica* and chemical competent cells of *E. coli* were prepared by standard protocols. IPTG and mDAP were used at a final concentration of 1 mM. Agar (used at 1.5%) and agarose (used at 1%), purchased from Invitrogen. T4-DNA ligase and *HindIII* from New England Biolabs. All other chemicals were purchased from Applichem, unless otherwise specified.

## 3. Methods

An empty plasmid, pCM699, was derived from the plasmid, pCM655/DapE [13], by deleting the DapE gene by *HindIII* digestion and religation of the vector backbone (Table 1). The strain TN5911 (chloramphenicol resistance) is a knockout strain for DapE and for several dipeptidases; therefore, mDAP has to be supplemented in both minimal and enriched media. An appropriate source of leucine and proline is also required in minimal medium and supplementation with lysine provides better growth (Prof. Miller; personal communication). Two strains were derived from the knockout-strain TN5911 by transforming the plasmids pCM655/dapE and pCM699 (strains TN5935 and TN5959, resp.). The plasmids (pCM655/dapE and pCM699) were transformed into TN5911 by electroporation (1.8 kV, 0.1 cm cuvettes) and recovered with 1 mL of SOC medium containing mDAP and chloramphenicol for 1 hour at 37°C in a shaking incubator and selected for plasmid encoded ampicillin resistance. A single colony of each strain TN5935 and TN5959 was picked and cultured in 5 mL of LB containing appropriate amounts of chloramphenicol, ampicillin, and mDAP over night at 37°C in a shaking incubator. The cultures of the two strains were diluted by 10 times (to dilute-out any remaining mDAP) and each strain was plated in a minimal medium supplemented or not with mDAP. In a similar manner,* E. coli* strains, TN5960 and TN5962, were derived from wild-type XL1-Blue by transforming the plasmid pCM699 and pCM655/DapE, respectively. Analysis of soluble cell extracts of TN5960 and TN5962 by SDS-PAGE revealed the presence of an additional species of about 42 KDa in cells harbouring pCM655/DapE (i.e., strain TN5962), corresponding to the expected size of the recombinant *S. enterica* DapE in *E. coli*.

To test l-captopril inhibition, appropriate dilutions of each strain (e.g., TN5935 and TN5959) were spread on selection plates with and without mDAP. After spreading the cultures, sterile paper discs soaked in several concentrations of l-captopril were placed on each plate; alternatively, a given amount of compound was placed in powder form directly at a defined site on the agar, such that it could be covered by a paper disk, and subsequently 10 *μ*L of sterile water were added carefully on the disk. These selection plates were incubated overnight at 37°C.

## 4. Results

**Figure 1:**
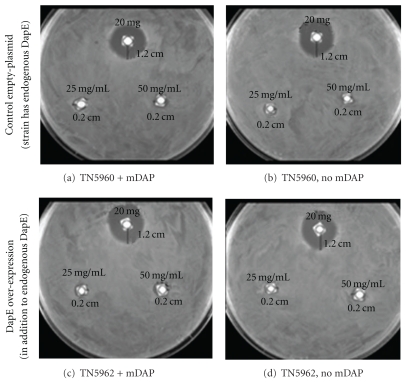
DapE independent inhibition of *E. coli* by l-captopril. (a) The strain has constitutively expressed endogenous *dapE* gene, and the medium was also supplemented with mDAP; (b) the strain has constitutively expressed endogenous *dapE* gene but the medium was not supplemented with mDAP; (c) the strain has overexpressed plasmid encoded DapE (in addition to the constitutively expressed endogenous DapE) and the medium was also supplemented with mDAP; (d) the strain has overexpressed plasmid encoded DapE (in addition to the constitutively expressed endogenous DapE) but the medium was not supplemented with mDAP.

First, we compared the growth of *E. coli* in the presence of l-captopril, with and without mDAP supplementation in the agar-medium, using a disk-diffusion assay (Figure 1 and Table 2). We confirmed the modest inhibitory activity of l-captopril in *E. coli*, as previously reported. However, to our surprise, l-captopril antimicrobial activity was independent of mDAP supplementation in *E. coli*. Moreover, the extent of inhibition was also unaffected by heterologous expression of DapE (from *Salmonella enterica*) in *E. coli *(Figures 1(c) and 1(d)).

Second, considering that overexpression of DapE from *S. enterica* in *E. coli* did not affect l-captopril inhibition, we set out to confirm that the DapE plasmid was functional for DapE expression. As previously reported [13], the DapE knock-out strain (TN5911) of *S. enterica* only grows when harbouring the plasmid carrying *dapE* gene (pCM655/DapE, within strain TN5935) or by supplementing the medium with mDAP, which is derived from the native product of DapE hydrolytic activity, l,l-DAP. Consequently, strain TN5935 (which harbours a plasmid-encoded *dapE* gene) grew even in the absence of mDAP supplementation, since it can produce its own DAP for cell-wall synthesis. As negative control, we corroborated that the knockout strain harbouring the corresponding “empty plasmid” (conferring ampicillin resistance but with no *dapE* gene; strain TN5959) did not grow in absence of mDAP supplementation. 

**Figure 2:**
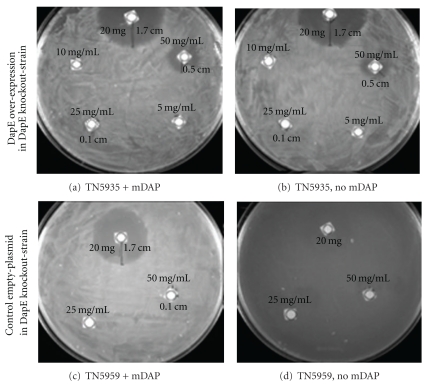
DapE independent inhibition of *Salmonella enterica* by l-captopril. (a) The knockout-strain has plasmid encoded *dapE* gene, and the medium is also supplemented with mDAP; (b) the knockout-strain has plasmid encoding *dapE* gene but no mDAP supplemented in the medium; (c) the knockout-strain has no plasmid encoding *dapE* gene but the medium was supplemented with mDAP; (d) the knockout-strain has no plasmid encoding *dapE* gene and no mDAP was supplemented.

Third, we tested the growth inhibiting-activity of l-captopril in these various recombinant strains of *S. enterica*. We hypothesised that if l-captopril was inhibiting DapE *in bacteria, *then mDAP supplementation or DapE overexpression would alleviate the antimicrobial effects of the drug. Surprisingly, l-captopril inhibited the growth of TN5959 (harbouring an empty plasmid) despite the addition of mDAP (Figure 2 and Table 3). We also tested whether the modest l-captopril inhibition could be overcome by DapE overexpression. Here also, we were surprised to find that similar l-captopril inhibition of strain TN5935 (overexpressing DapE), even when mDAP was additionally supplemented in the medium.

The zone of inhibition was slightly more in strain TN5935 compared to TN5959, although this difference was very subtle and could only be seen when the paper disks were soaked at the concentrations 25 mg/mL and 50 mg/mL, but not when 20 mg were added (Table 3); at the latter amount of compound, the extent of inhibition was significant and identical to that observed in TN5959. We speculate that this marginally higher l-captopril inhibition in the strain overexpressing DapE (TN5935) compared with TN5959 is due to the high metabolic load of the cell caused by overexpression of DapE.

Taken together, these data strongly suggest that l-captopril modestly inhibits both *S. enterica* and *E. coli *to a similar extent, but in a DapE-independent manner.

## 5. Discussion

Diaminopimelic acid is an essential precursor for cell wall synthesis in many bacteria, including *E. coli *and *S. enterica * [5, 15]. Inhibition of DapE from *H. influenzae in vitro* and inhibition in *Escherichia coli* by l-captopril *in bacteria* was recently reported [3]. In the process of enzymatically characterizing mutant variants of *Salmonella enterica* DapE, we decided to test the inhibition of DapE with l-captopril *in bacteria*. We hypothesised that if l-captopril was inhibiting bacterial growth by inhibiting DapE, then mDAP supplementation in the growth media would overcome drug-inhibition. However, to our surprise we found that l-captopril inhibits the bacterial strains (*S. enterica* and *E. coli*) in a DapE-independent manner.

The putative substrate binding site and the metal coordinating residues are very much conserved in the DapE of *E. coli*, *S. enterica*, and *H. influenzae, *all of which are thought to have similar binuclear metal-centres and identical catalytic mechanism [4, 7–9]. Consequently, considering such structural and functional conservation among DapE from these various bacteria, it would be expected that l-captopril is able to inhibit the different homologs in a similar manner, proving potentially active as a broad-selectivity antibiotic. It is noteworthy that different isoforms of DapE (e.g., from different organisms or with different metal contents) could be inhibited differently, thereby making it difficult to find a single broad-spectrum antibiotic targeting DapE. Our data show that l-captopril is not a broad-spectrum antibiotic targeting DapE in bacteria, because it neither targets DapE in *E. coli* nor in *S. enterica*. Since the extent of inhibition by l-captopril *in bacteria* was similar in all of our experiments in *E. coli* and *S. enterica* and independent of DapE, we suggest that the modest antimicrobial activity of this compound is probably due to inhibition of hitherto-unidentified metalloproteins other than DapE.

In summary, our results show that despite the reported inhibition of DapE from *H. influenzae* by l-captopril *in vitro*, it is very unlikely that DapE inhibition contributes to any significant antimicrobial activity in Gram-negative bacterial cultures. Therefore, l-captopril is a modest antibiotic, inhibiting Gram-negative bacteria at high doses, but its mechanism of action or molecular target remains unknown. 

Finally, considering that DapE is a promising antibiotic target, the failure of a lead-compound that inhibits DapE *in vitro* to show any measurable anti-DapE effect *in bacteria *provides a sobering reminder of the difficulty of translating *in vitro* data to effects *in vivo,* even in pure microbiological cultures. However, despite our finding that captopril does not lead to DapE inhibition in bacteria, the development of other (more effective) DapE inhibitors *in vitro* and *in vivo* continues to be a very worthy goal and a promising line of research toward new antibiotics. We suggest that mDAP supplementation, as described here, will offer a facile and very robust method for confirming the selectivity of novel antibiotics targeting DapE *in bacteria*.

## Figures and Tables

**Table 1:** Summary of strains and plasmids used in this work.

Plasmids	Genotype	Remarks
pCM655/ DapE	Plasmid with *dapE* gene	Allows overexpression of DapE, thus rescuing DapE knockout strains (even in absence of mDAP supplementation). Also confers ampicillin-resistance
pCM699	Plasmid without *dapE* gene	Confers ampicillin-resistance, but does not genetically rescue DapE knockout strains

*S. enterica* strains		
TN5911	*dapE* knockout strain	Strain does not grow in absence of mDAP supplementation
TN5935	TN5911 + pCM655/DapE	DapE is overexpressed from plasmid and strain can grow in absence of mDAP supplementation
TN5959	TN5911 + pCM699	Plasmid does not contain DapE and knockout strain does not grow in absence of mDAP supplementation

*E. coli* strains		
XL1-Blue	Wildtype (from Stratagene)	Has endogenous DapE and can grow in absence of mDAP supplementation
TN5960	XL1-Blue + pCM699	Plasmid does not contain DapE, but strain can grow in absence of mDAP supplementation due to endogenous DapE
TN5962	XL1-Blue + pCM655/DapE	In addition to endogenous DapE, this strain has plasmid-encoded DapE overexpression (from *S. enterica*).

**Table 2:** Inhibition of *E. coli* growth by l-captopril.

Strain with endogenous DapE	TN5960 (Plasmid without *dapE* gene)		TN5962 (Plasmid with *dapE* gene)	
Supplemented with mDAP	Yes	No	Yes	No
	Radius of zone of inhibition by l-captopril			

25 mg/mL	0.2 cm	0.2 cm	0.2 cm	0.2 cm
50 mg/mL	0.2 cm	0.2 cm	0.2 cm	0.2 cm
20 mg	1.2 cm	1.2 cm	1.2 cm	1.2 cm

**Table 3:** Inhibiton of *Salmonella enterica* growth by l-captopril.

DapE-knockout strains	TN5935 (Plasmid with *dapE* gene)		TN5959 (Plasmid without *dapE* gene)	
Supplemented with mDAP	Yes	No	Yes	No
	Radius of zone of inhibition by l-captopril			

25 mg/mL	0.1 cm	0.1 cm	0.0 cm	No growth
50 mg/mL	0.5 cm	0.5 cm	0.1 cm	No growth
20 mg	1.7 cm	1.7 cm	1.7 cm	No growth
